# *Undaria pinnatifida* (Wakame) Intake Ameliorates High-Fat Diet-Induced Glucose Intolerance via Promoting GLUT4 Expression and Membrane Translocation in Muscle

**DOI:** 10.1155/2023/9774157

**Published:** 2023-01-10

**Authors:** Motoki Imai, Fumitaka Kawakami, Mutsumi Chiba, Makoto Kanzaki, Hiroko Maruyama

**Affiliations:** ^1^Department of Cytopathology, Graduate School of Medical Sciences, Kitasato University, Sagamihara 252-0373, Japan; ^2^Department of Regulation Biochemistry, Graduate School of Medical Sciences, Kitasato University, Sagamihara 252-0373, Japan; ^3^Department of Biomedical Engineering, Graduate School of Biomedical Engineering, Tohoku University, Sendai 980-8579, Japan

## Abstract

Type 2 diabetes mellitus (T2DM), a lifestyle-related disease, is developed due to eating habits and decreased physical activity. Diabetes also increases the risk of cancer and major neurodegenerative diseases; controlling the onset of diabetes helps prevent various illnesses. Eating seaweed, such as *Undaria pinnatifida* (wakame), is a part of the Asian food culture. Therefore, we analyzed the antidiabetic effect of wakame intake using the high-fat diet-induced diabetes mouse model. Furthermore, we analyzed the effect of wakame extract on the cell membrane translocation of glucose transporter-4 (GLUT4) and activation of insulin signal molecules, such as AKT and AMPK, in insulin-sensitive tissues. Differentiated C2C12 cells were incubated with wakame components. The membrane translocation of GLUT4 and phosphorylation of AKT and AMPK were investigated with immunofluorescence staining and Western blotting, respectively. Also, male C57BL/6J mice were fed the normal diet (ND), high-fat diet (HFD), ND with 1% wakame powder (ND + W), or HFD with 1% wakame powder (HFD + W). We evaluated the effect of wakame intake on high-fat diet-induced glucose intolerance using an oral glucose tolerance test. Moreover, we analyzed insulin signaling molecules, such as GLUT4, AKT, and AMPK, in muscle using Western blotting. GLUT4 membrane translocation was promoted by wakame components. Also, GLUT4 levels and AKT and AMPK phosphorylation were significantly elevated by wakame components in C2C12 cells. In addition, the area under the curve (AUC) of the HFD + W group was significantly smaller than that of the HFD group. Furthermore, the level of GLUT4 in the muscle was increased in the wakame intake group. This study revealed that various wakame components exerted antidiabetic effects on the mice on a high-fat diet by promoting glucose uptake in the skeletal muscle, enhancing GLUT4 levels, and activating AKT and AMPK.

## 1. Introduction

Type 2 diabetes mellitus (T2DM) is caused by a long-term glucose metabolism disorder characterized by obesity and glucose intolerance [1]. Lifestyles, such as overeating, lack of physical activity, dietary habits, and stress, are closely related to the onset of T2DM [2].

One of the characteristics of T2DM is abnormal intracellular glucose uptake in insulin-sensitive tissues which induces glucose intolerance [3]. Essentially, blood glucose concentrations are maintained appropriately by glycogenesis and cellular uptake of glucose. When blood glucose levels increase, insulin is secreted from pancreatic beta cells and acts on insulin-sensitive tissues such as skeletal muscle [4], liver [5], and adipose tissue [6], causing glucose to be taken up into the cells and blood glucose levels to decrease.

The insulin signaling pathways play a critical role in glucose tolerance [7]. Protein kinase B (AKT) is one of the critical molecules in the insulin signaling pathways in insulin-sensitive cells [8]. AKT is activated by insulin through the insulin receptor, insulin receptor substrate 1 (IRS-1), and phosphatidylinositol-3 kinase (PI3K). Activated AKT promotes glucose uptake by increasing the membrane translocation of GLUT4, encoded by Slc2a4, to the cell surface [9]. In addition, the activation of 5′ adenosine monophosphate-activated protein kinase (AMPK) promotes GLUT4 membrane translocation [10]. The activation of AMPK is induced by exercise and adiponectin, one of the adipocytokines [11]. Thus, the activation of AKT and AMPK induces GLUT4 vesicle translocation to the cell membrane and increases the level of GLUT4 at the cell surface, thereby promoting glucose uptake.

Seaweed is an essential ingredient in Japanese food culture. *Undaria pinnatifida* (wakame), a brown seaweed, is one of the most popular seaweeds and is used in traditional Japanese cuisine. Wakame includes several components, such as minerals, vitamins, dietary fiber, polyphenols, and peptides [12]. Wakame provides many benefits to organisms, including anticancer [13], antihypertensive [14, 15], and antimetabolic syndrome effects [16, 17]. Based on these reports, we hypothesized that wakame might effectively prevent the onset of T2DM, one of the metabolism-related diseases.

This study investigated the effect of the daily consumption of wakame on glucose tolerance in model mice with abnormal glucose metabolism induced by a high-fat diet. Also, we examined the effect of wakame on the molecules related to glucose uptake, such as AKT, AMPK, and GLUT4, in insulin-sensitive tissue such as the muscle. In addition, we investigated the effect of seaweed extracts on the membrane translocation of GLUT4, insulin signaling pathway, and glucose uptake using the C2C12 myotube cells derived from insulin-sensitive tissues.

## 2. Materials and Methods

### 2.1. Cell Culture

C2C12 cells were cultured in high-glucose (4500 mg/L) Dulbecco's Modified Eagle's Medium (DMEM; Sigma-Aldrich Co. LLC., St. Lois, MO, USA) with 10% fetal bovine serum (FBS; GIBCOTM, Thermo Fisher Scientific, MA, USA), 100 U/mL penicillin (Nacalai Tesque), and 100 *μ*g/mL streptomycin (Nacalai Tesque) at 37°C in a humidified atmosphere of 95% air and 5% CO_2_. The C2C12 cells were differentiated by seeding them in plates with high-glucose DMEM with 10% FBS medium until reaching 90% confluency and then inducing them in the differentiation medium, i.e., high-glucose DMEM with 2% calf serum. The medium was changed every 24 h. After the C2C12 cells were cultured in the differentiation medium for 120 h, the cells were cultured in a DMEM FBS-free medium containing wakame components (fucoidan [18], ethanol extract, hexane extract, fucoxanthinol [19], and wakame peptide [20]) and metformin (kind of positive control) for 24 h (Table 1). The cells were then washed 3 times with ice-cold PBS and harvested for the Western blot analysis of insulin signaling molecules. In addition, ethanol extract or hexane extract is extracted from the residue of wakame peptide extraction. Wakame peptide extracted residue (1 g dry weight) was soaked in 10 mL of ethanol or hexane with ultrasonication was performed for 30 min, centrifuged 3000 ×g, and the extract was filtered through cotton. The supernatant fraction was concentrated and vacuum dried. The dried product was dissolved in DMSO and used as ethanol extract or hexane extract.

### 2.2. Immunofluorescent Cytochemistry (IFC) of C2C12 Cell

Myc-GLUT4-ECFP-expressing stable C2C12 cells, established by Dr. Makoto Kanzaki, were cultured and differentiated in 24-well plates onto glass-bottom dishes (No. 1 S thickness, 0.16–0.19 mm; Matsunami Glass, Osaka, Japan). After differentiation, the cells were treated with the KRPH buffer and stimulated with wakame components for 20 min. IFC was performed as described previously [21].

### 2.3. Animal Experiments

Seven-week-old C57BL/6J male mice were purchased from CLEA Japan, Inc. (Tokyo, Japan), and allowed ad libitum access to a normal diet (ND, AIN-93M: 15% kcal from fat, Oriental Yeast Co., Ltd., Tokyo, Japan), ND supplemented with 1% of the indicated wakame powder (ND + W), high-fat diet (HFD) with 60% of the calories from fat (HFD-60) (Oriental Yeast Co., Ltd.), or HFD supplemented with 1% of the indicated wakame powder (HFD + W) (Riken Vitamin Co. Ltd., Tokyo, Japan) for 3 months with ad libitum access to experimental diet and water. These mice were maintained, with two or three mice per cage, at 22°C, 60% humidity, and a 12 h light and dark cycle under specific pathogen-free conditions. This animal experiment has been approved by the Animal Experiment Committee of the Faculty of Hygienic Sciences, Kitasato University.

### 2.4. Oral Glucose Tolerance Test (OGTT)

An oral glucose tolerance test (OGTT) was performed three months after exposure to the experimental diets. After fasting for 16 h from the day before the OGTT test, blood was collected from the mouse's tail vein to measure the fasting blood glucose using the Glutest Neo Alpha glucometer (Sanwa Chemical Institute, Japan). A 16% glucose solution was then orally administered to the mice with gavage at a dose of 2 g/kg body weight. Blood was collected from the tail vein at 15, 30, 45, 60, 90, and 120 min after glucose administration to measure the blood glucose level. Glucose tolerance was evaluated by the area under the curve (AUC) from the change in blood glucose concentration after the oral administration of 16% glucose.

### 2.5. Western Blot

The skeletal muscle tissue samples collected from the mice and the cultured cell proteins were isolated using RIPA buffer. Western blot was performed as described previously [21]. The phosphor-specific antibodies, P-AKT (S473, 4051S; Cell Signaling Technology) and P-AMPK (T172, 2531S; Cell Signaling Technology), were used at 1 : 1000 dilution. The total antibodies, GLUT4 (2213S; Cell Signaling Technology), AKT (9272S; Cell Signaling Technology), AMPK (2793S; Cell Signaling Technology), *β*-actin (#12004164; BIO-RAD), cyclophilin B (ab16045; Abcam), and GAPDH (2537S; Cell Signaling Technology), were used at 1 : 2000. Secondary antibodies, IRDye® 800CW Goat anti-Rabbit (LI-COR), IRDye® 680RD Goat anti-Mouse (LI-COR), HRP Donkey anti-Mouse IgG (H + L) (Jackson ImmunoResearch), and HRP Donkey anti-Rabbit IgG (H + L) (Jackson ImmunoResearch), were used at 1 : 10,000.

### 2.6. Statistical Analysis

The data were analyzed with the GraphPad Prism 8 software (GraphPad Software, CA, USA). All the data were expressed relative to the control values and presented as mean ± standard deviation (SD). *p* values were calculated using the Tukey test; a *p* value of less than 0.05 was considered statistically significant.

## 3. Results

### 3.1. Wakame Components Promote GLUT4 Membrane Translocation in C2C12-G4 Myotube Cells

**Figure 1:**
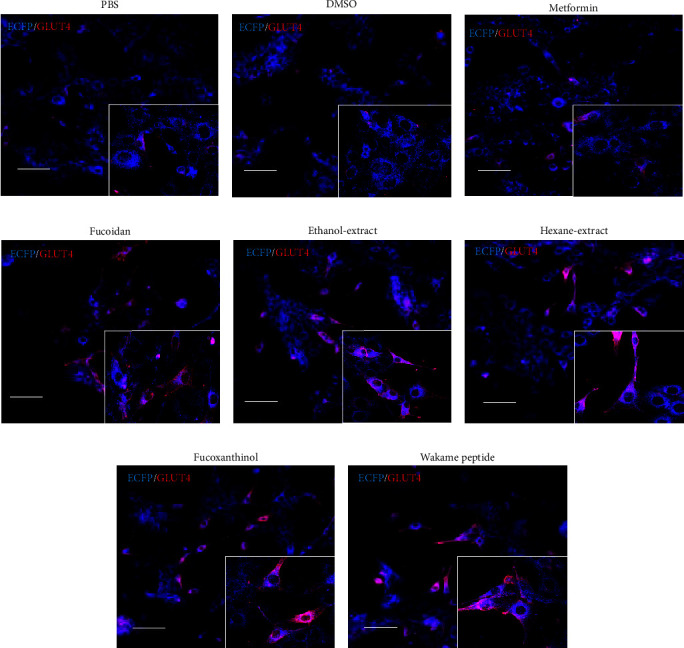
Effect of wakame components on GLUT4 membrane translocation in C2C12 cells. A representative image shows the translocated GLUT4 (red) and endogenous GLUT4 (blue). Myc-GLUT4-ECFP-expressing stable C2C12 (C2C12-G4) cells were treated with (a) PBS, (b) DMSO, (c) 1 mM metformin, (d) 1 mg/mL fucoidan, (e) 50 *μ*g/mL ethanol extract, (f) 50 *μ*g/mL hexane extract, (g) 1 *μ*g/mL fucoxanthinol, or (h) 100 *μ*g/mL wakame peptide for 20 min. The C2C12-G4 cells were then fixed with 4% PFA and stained with anti-Myc antibody followed by Alexa594-labeled secondary antibody. Scale bar, 50 *μ*m.

We observed the effects of wakame components on the cell membrane translocation of GLUT4 using ECFP-GLUT4-Myc expressing stable C2C12-G4 myotube cells. The cells were observed after stimulation with PBS or wakame components for 30 min. The translocation of GLUT4 to the cell membrane was not promoted in C2C12-G4 myotube cells stimulated with PBS, DMSO, or metformin. However, GLUT4's translocation to the membrane in the C2C12-G4 cells was stimulated by wakame components, such as fucoidan, ethanol extract, hexane extract, fucoxanthinol, and wakame peptide (Figures 1(a)–1(h)). In addition, GLUT4 translocation in C2C12-G4 myotube cells was promoted by stimulation of wakame components such as fucoidan, ethanol extract, hexane extract, fucoxanthinol, and wakame peptide.

### 3.2. Wakame Components Regulate GLUT4 Levels at the Membrane and AKT and AMPK Activation in C2C12 Myotube Cells

**Figure 2:**
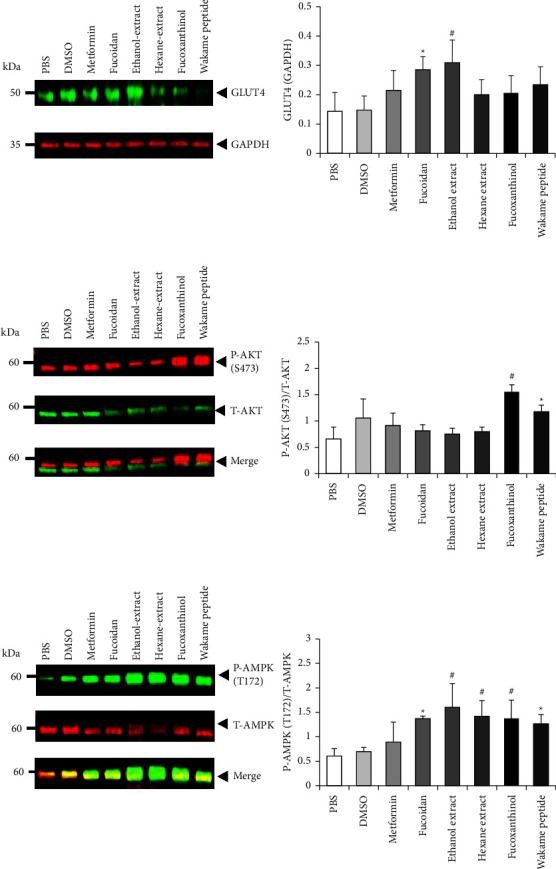
Effect of wakame components on AKT and AMPK phosphorylation and GLUT4 levels in C2C12 cells. Differentiated C2C12 cells were treated with PBS, DMSO, metformin (1 mM), fucoidan (1 mg/mL), ethanol extract (50 *μ*g/mL), hexane extract (50 *μ*g/mL), fucoxanthinol (1 *μ*g/mL), or wakame peptide (100 *μ*g/mL) for 24 h before lysis. The lysates were analyzed with Western blot using (a) GLUT4, (b) P-AKT (s473) and T-AKT, and (c) P-AMPK (T172) and T-AMPK antibodies and quantified. The level of GLUT4 was normalized by that of GAPDH. The intensity of P-AKT and P-AMPK bands was normalized by that of their respective total protein bands. The data are presented as mean ± SD (*n* = 4). The data were analyzed with one-way ANOVA combined with the Tukey test. ^*∗*^*p* < 0.05, compared to PBS; #*p* < 0.05, compared to DMSO.

We investigated the effect of wakame extracts on AKT and AMPK activation and GLUT4 levels in C2C12 cells. The translocation of GLUT4 in C2C12-G4 myotube cells to the cell surface could be confirmed by IFC. The intracellular level of GLUT4 protein was significantly increased by adding fucoidan or the ethanol extract and hexane extract; however, fucoxanthinol and wakame peptide did not affect the level of GLUT4 in C2C12 cells (Figure 2(a)). On the other hand, the phosphorylation of AKT was promoted by fucoxanthinol and the wakame peptide (Figure 2(b)). Furthermore, the phosphorylation of AMPK in C2C12 cells was significantly increased by fucoidan, ethanol extract, and hexane extract, fucoxanthinol, and wakame peptide (Figure 2(c)).

### 3.3. Wakame Improves the Glucose Intolerance of HFD-Induced Mice

Wakame components were observed to promote the cell membrane translocation of GLUT4 by activating AKT and AMPK and increasing the intracellular level of GLUT4. Therefore, an experiment was carried out to clarify how wakame intake affected glucose uptake in vivo using diabetic mice induced by a high-fat diet.

**Figure 3:**
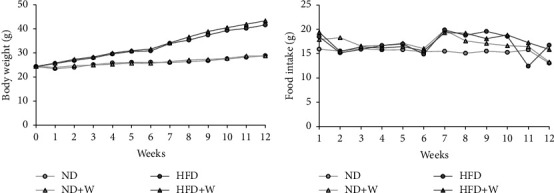
The effect of wakame on body weight and food consumption. Seven-week-old C57BL/6J mice were reared on experimental diets for 3 months and measured for body weight and food consumption weekly. (a) The change in body weight over 3 months. (b) The weekly food consumption over 3 months.

**Figure 4:**
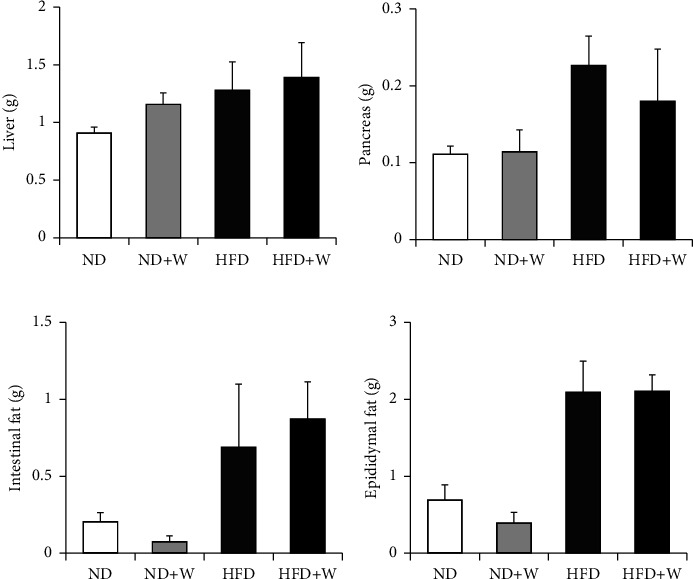
Weight comparison of insulin-sensitive organs and tissues in mice on different experimental diets. Seven-week-old C57BL/6J mice were reared on experimental diets for 3 months. Then, the organs and tissues from the ND, ND + W, HFD, and HFD + W mice were dissected, collected, and measured after 16 h of fasting. (a) Liver. (b) Pancreas. (c) Intestinal fat. (d) Epididymal fat. Data are presented as mean ± SD (*n* = 4-5 per group).

**Figure 5:**
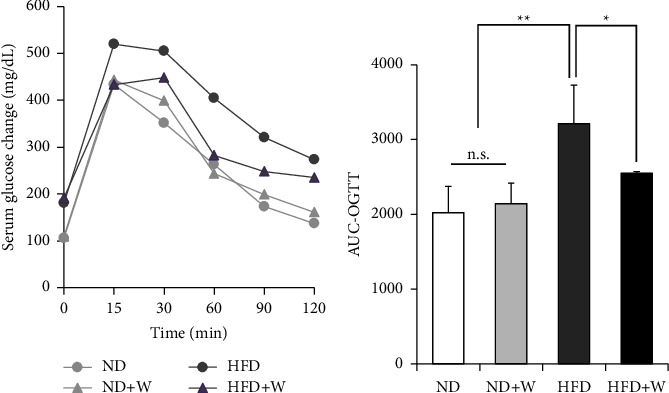
Comparison of serum blood glucose change in OGTT of mice taking experimental diet. Seven-week-old C57BL/6J mice were reared on experimental diets for 3 months. (a) Oral glucose tolerance test (OGTT, 2 g/kg) at 0, 15, 30, 45, 60, 90, and 120 min. (b) The change in the area under the curve (AUC) value of the serum glucose in the mice during the OGTT. Data are presented as mean ± SD (*n* = 4-5 per group). The data were analyzed with one-way ANOVA combined with the Tukey test. ^*∗*^*p* < 0.05; ^*∗∗*^*p* < 0.01.

We investigated how wakame affected obesity and diabetes by studying the body weight gain and food intake of the mice on a high-fat diet supplemented with wakame. There was no difference in weight gain and food intake between the control groups, ND and HFD, and the experimental groups, ND + W and HFD + W (Figures 3(a) and 3(b)). The effect of wakame on glucose tolerance was assessed with the oral glucose tolerance test (OGTT). OGTT was performed after the mice were on the experimental diets for 3 months. Also, the liver, pancreas, intestinal fat, and epididymal fat of the mice were examined; there was no difference between ND group and ND + W group or HFD group and HFD + W group (Figures 4(a)–4(d)). AUC values were significantly increased in HFD group compared to ND group. The AUC values were also significantly lower in HFD + W group compared to HFD group. However, there was no significant difference between the ND group and ND + W groups (Figure 5(b)). Furthermore, there was no significant difference in fasting blood glucose levels between ND group and ND + W group or HFD group and HFD + W group (Figure 5(a)).

### 3.4. Wakame Promotes Glucose Uptake by Increasing GLUT4 Levels in the Skeletal Muscle

**Figure 6:**
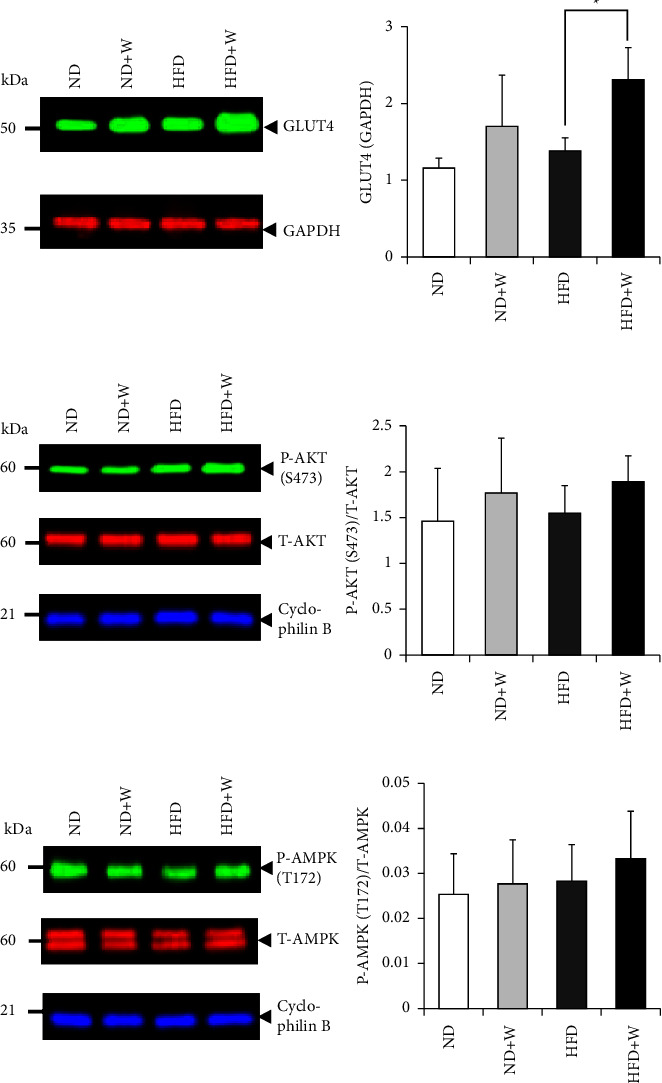
Wakame's effect on level and phosphorylation of insulin signaling molecules and GLUT4 in skeletal muscle. The skeletal muscle cells were collected via dissection from the ND, ND + W, HFD, and HFD + W mice. The cell lysates were analyzed by Western blotting with (a) GLUT4, (b) P-AKT (s473) and T-AKT, and (c) P-AMPK (T172) and T-AMPK antibodies. The level of GLUT4 was normalized to that of GAPDH. The level of P-AKT and T-AMPK was normalized to that of their respective total protein. The data were presented as mean ± SD (*n* = 4-5) and analyzed with one-way ANOVA combined with the Tukey test. ^*∗*^*p* < 0.05.

Skeletal muscle has a physiologically important role in glucose uptake and insulin sensitivity [22, 23]. On the other hand, GLUT4 is an insulin-dependent glucose transporter that plays a crucial role in glucose uptake via insulin signaling [24]. Therefore, we investigated the change in the level of GLUT4 in the skeletal muscle in the mice treated with wakame. The level of GLUT4 in the skeletal muscle was significantly increased in the wakame-treated HFD + W group compared to the HFD groups (Figure 6(a)).

Since AKT and AMPK activation also plays a critical role in intracellular glucose uptake in muscle cells, we examined the phosphorylation of Ser473 of AKT and Thr172 of AMPK in the skeletal muscle. The ratio of the phosphorylated AKT to the total AKT was not significantly different between the control, ND, and HFD groups and the experimental ND + W and HFD + W groups (Figure 6(b)). Also, the ratio of phosphorylated AMPK to the total AMPK was not significantly different between the control ND and HFD groups and the experimental ND + W and HFD + W groups (Figure 6(c)).

## 4. Discussion

In this study, we investigated the effect of wakame components on glucose intolerance. First, we examined the effect of wakame components on the level of GLUT4, a molecule related to intracellular glucose uptake, and the activation of AKT and AMPK. We found that the addition of wakame component promoted the translocation of GLUT4 to the cell membrane and increased the level of GLUT4. AKT and AMPK were also activated by wakame component. Thus, wakame component was considered to promote intracellular glucose uptake and improve glucose tolerance.

We also investigated the effect of wakame intake on glucose intolerance in mice. We found that glucose intolerance was exacerbated in the mice on the high-fat diet but was suppressed in the mice on the high-fat diet with wakame. In addition, GLUT4, the factor related to intracellular glucose uptake, was increased in the skeletal muscle in the mice on high-fat diet with wakame.

It has been reported that intracellular glucose uptake promotes the activity of seaweed components, especially fucoxanthin, a carcinoid pigment in brown algae [25, 26]. In this experiment, the fucoxanthinol, a metabolite of fucoxanthin, and a peptide with a high content in wakame (wakame peptide) [27], ethanol-extracted, and hexane-extracted were used to investigate the effect of wakame on intracellular glucose uptake in C2C12 cells. First, we investigated the translocation of GLUT4 to the membrane and found that the ethanol-extracted component, hexane-extracted component, fucoidan, fucoxanthinol, and the wakame peptide promoted the translocation of GLUT4 to the cell membrane. Thus, the wakame extracts likely affected the AKT and AMPK signaling involved in GLUT4 membrane translocation.

We also found that the addition of fucoxanthinol and wakame peptide promoted the phosphorylation of AKT. In cell culture experiments with insulin, fucoxanthinol, a metabolite of fucoxanthin, promotes AKT phosphorylation by increasing insulin receptor expression in soleus muscle [25]. However, since insulin was not added under our experimental conditions, fucoxanthinol and the wakame peptide likely activated AKT without acting through the insulin receptors. It was clarified that AMPK was activated by the ethanol-extracted component and hexane-extracted component, which were thought to be extracted mainly from the fat-soluble components of wakame. Furthermore, fucoidan, fucoxanthinol, and wakame peptide were found to activate AMPK. It has been reported that the activation of AMPK promotes GLUT4 expression and membrane translocation to increase intracellular glucose uptake [10]. Here, wakame components were found to also elevate GLUT4 levels and glucose uptake through the activated AMPK. Thus, the seaweed components likely increase GLUT4 levels and glucose uptake through the activation of AMPK in muscle cells.

The activation of the glucose uptake signals and increased levels of GLUT4 by the seaweed components in cultured muscle cells were observed, suggesting that the ingestion of wakame exerted an antidiabetic effect on the body. Therefore, we investigated the effect of wakame intake on impaired glucose tolerance due to a high-fat diet. Glucose intolerance was increased in the mice that were fed a high-fat diet, as indicated by their large AUC values in the OGTT. On the other hand, the intake of wakame suppressed abnormal glucose tolerance.

The activation of the molecules related to intracellular glucose uptake by wakame was considered. Therefore, we analyzed the effects of wakame intake on skeletal muscles responsible for most of the glucose metabolism in the body. The intake of wakame was found to increase the level of GLUT4 in skeletal muscle significantly. Still, the phosphorylation of AKT and AMPK was not significantly enhanced by the intake of wakame. These results suggest that the intake of wakame increases the level of GLUT4 in skeletal muscle and suppresses the abnormal glucose tolerance induced by a high-fat diet.

The present study has uncovered that wakame components exert antidiabetic effects on the mice on a high-fat diet by promoting glucose uptake in skeletal muscle by elevating the level of GLUT4 and activating AKT and AMPK. These results suggest that the daily intake of wakame has a preventive and ameliorating effect on diabetes. However, this study has not identified the active components in wakame. Therefore, we need to identify the active ingredients in wakame in future studies. Furthermore, in this study, we demonstrated that wakame components directly acted with cultured cells. However, since wakame components may be affected by digestion and absorption in vivo, further studies on the effects of wakame components are required in the future.

## 5. Conclusion

We revealed the antidiabetic effects of extracted components of wakame on the mice on a high-fat diet. These components promoted glucose uptake in the skeletal muscle, enhancing GLUT4 levels and activating AKT and AMPK. These results suggest that intake of wakame has a preventive and ameliorating effect on diabetes and is expected to help prevent cancer and neurodegenerative diseases caused by diabetes.

## Figures and Tables

**Table 1:** Seaweed components used in cell culture experiment.

Sample name	Final concentration	Solvent
Metformin	1 mM	PBS
Fucoidan	1 mg/mL	PBS
Ethanol extract	50 *μ*g/mL	DMSO
Hexane extract	50 *μ*g/mL	DMSO
Fucoxanthinol	1 *μ*g/mL	DMSO
Wakame peptide	100 *μ*g/mL	PBS

## Data Availability

The data used to support the findings of this study are included within the article.
